# *Trichoderma harzianum* T-22 Induces Systemic Resistance in Tomato Infected by *Cucumber mosaic virus*

**DOI:** 10.3389/fpls.2016.01520

**Published:** 2016-10-10

**Authors:** Antonella Vitti, Elisa Pellegrini, Cristina Nali, Stella Lovelli, Adriano Sofo, Maria Valerio, Antonio Scopa, Maria Nuzzaci

**Affiliations:** ^1^School of Agricultural, Forestry, Food and Environmental Sciences, Università degli Studi della Basilicata, PotenzaItaly; ^2^Department of Agriculture, Food and Environment, University of Pisa, PisaItaly

**Keywords:** disease control, host-pathogen-antagonist interaction, *Solanum lycopersicum* var. *cerasiforme*, *Cucumber mosaic virus*, *Trichoderma harzianum* strain T-22, photosynthetic performance, reactive oxygen species scavenging enzymes, phytohormones

## Abstract

Understanding the induction of plant defenses against viruses using biocontrol agents is essential for developing new strategies against these pathogens, given the ineffectiveness of chemical treatments. The ability of *Trichoderma harzianum*, strain T-22 (T22) to control *Cucumber mosaic virus* (CMV) in *Solanum lycopersicum* var. *cerasiforme* plants and the changes in the physiology of tomato treated/infected with T22/CMV were examined. Plant growth-promoting effects, photosynthetic performance, reactive oxygen species scavenging enzymes, and phytohormones were investigated. T22 improved tomato growth in terms of plant height and improved photosynthesis, total chlorophyll content and plant gas exchange. In contrast, CMV induced a negative effect on dry matter accumulation and inhibited the photosynthetic capacity. The analysis of plant hormones demonstrated that treating with T22 before or simultaneously to CMV infection, led to a systemic resistance by jasmonic acid/ethylene and salicylic acid signaling pathways. Conversely, systemic resistance was abscissic acid-dependent when T22 treatment was administered after the CMV infection. In conclusion, the data reported here indicate that the T22-based strategy may be the most effective measure against CMV.

## Introduction

The ability of viruses to significantly hinder the physiological processes of plants is closely related to a range of symptoms. *Cucumber mosaic virus* (CMV, family *Bromoviridae*, genus *Cucumovirus*), the plant virus with the largest host range of all RNA viruses (Edwardson and Christie, 1991), can cause a wide range of symptoms, such as yellow mottling, distortion and plant stunting, thus causing serious economic losses (Whitham et al., 2006). CMV genomic RNA 2 encodes the viral 2b protein, which is an important virulence determinant of the virus and is also responsible for the (i) interference of plant defense pathways and (ii) inhibition of salicylic (SA) and jasmonic (JA) acids-mediated resistance (Mochizuki and Ohki, 2012).

As a response to diseases, plants generally compensate with a broad range of cellular processes: (i) up- or down-regulation of specific genes; (ii) changes in the level of various substances implicated in the plant defense pathway, such as reactive oxygen species (ROS); (iii) activation of specific transcription factors, defense-regulated genes, heat-shock proteins; (iv) enhancement of the transport of macromolecules, enzymes, and phytohormones involved in defense signaling pathways [e.g., SA, JA, ethylene (ET), auxins (such as indole-3-acetic acid, IAA), cytokinins (CKs), abscisic acid (ABA), and gibberellic acid (GA)] (Bari and Jones, 2009; Vitti et al., 2013).

*Trichoderma* spp. are well-known as biocontrol agents (BCAs) due to their ability to antagonize plant pathogens (Benítez et al., 2004; Harman, 2006), to induce plant defense responses against pathogens, with beneficial effects on plant growth and development (Harman et al., 2004), and also to improve photosynthetic efficiency and respiratory activity, by reprogramming plant gene expression (Shoresh et al., 2010). Mastouri et al. (2010) demonstrated that, when tomato seeds were treated with *Trichoderma harzianum* strain T-22 (T22), a range of biotic and abiotic stresses were alleviated. This is because after the colonization of roots, *Trichoderma* spp., are able to chemically communicate with the plant and, sometimes, they act as endophytic symbionts. They are thus capable of correctly altering the expression of various plant genes and, as a consequence, plant physiology (Harman et al., 2012).

To date, little information is available regarding the effects of *Trichoderma* spp. in the induction of plant defenses against viruses (Lo et al., 2000; Luo et al., 2010; Elsharkawy et al., 2013). In addition - to the best of our knowledge - there are no studies on changes in plant physiology under treatment with *Trichoderma* spp. in the plant-virus pathosystem.

The effects of T22 on the induction of defense against CMV in *Solanum lycopersicum* var. *cerasiforme* (tomato) have been previously investigated by analyzing various biochemical and molecular aspects involved in the plant-virus interactions and demonstrating the clear involvement of ROS (Vitti et al., 2015). In order to study the mechanisms involved in this intricate three-way cross-talk among the plant, virus, and antagonist, this paper thoroughly analyzed the plant growth-promoting effect of *Trichoderma*, the photosynthetic performance of the plant, as well as the role of ROS scavenging enzymes and hormones in the regulation of tomato-CMV-T22 interaction. The possible use of the BCA T22 against CMV infection could be crucial not only for the development of an integrated understanding of the plant-pathogen-BCA complex, but also for developing rational strategies to manage viral diseases.

## Materials and Methods

### Trichoderma and CMV Sources

*Trichoderma harzianum* strain T-22 (T22) was used as a granule commercial formulation (Trianum G, Koppert, Berkel en Rodenrijs, The Netherlands), and *Cucumber mosaic virus* strain Fny (CMV-Fny) was utilized as purified. Details are reported in Vitti et al. (2015). The commercial formulation was controlled and characterized before starting the trials, as follows: serial dilutions of the granules of Trianum G (as such and also sterilized by autoclaving for 20 min at 121°C) in sterile water were carried out; aliquots of each suspension obtained were distributed on solid substrate (PDA+Agar) and in two liquid substrates (PDA and NaCl solution) and left to incubate at 25°C for 3–5 days. The isolation and characterization showed the presence of only T22 exclusively by material as such, but nothing from that autoclaved. At the same time, the roots of tomato seedlings grown on three different substrates (agar nutrient medium, gravel and sterile soil) were colonized with T22 and the colonization has been checked and detected in all cases. To be as close as possible to the field conditions, it was chosen to use the soil. Moreover, throughout the study, the colonization was checked, too. These remarks were considered as a starting point of the study.

### Plant Material and Experimental Design

After surface sterilization (using 1% Na-hypochlorite solution for 1 min), seeds of *Solanum lycopersicum* var. *cerasiforme* cv. Cherry were germinated on water-dampened filter paper in sterile Petri dishes at 26°C in an incubator for 2–3 days. One day after germination, seedlings were transferred to sterilized soil-filled pots, kept in a growth chamber with a 16-h photoperiod, at 26/23°C (day/night), and watered with ¼-strength Hoagland solution, as previously described (Vitti et al., 2015). Treatments with T22 were performed by incorporating Trianum G granules into the substrate used for planting (750 g m^-3^), following the application and the dose suggested by the manufacturer. CMV-Fny purified was used to mechanically inoculate tomato plants at the four-leaf stage (5 μg cotyledons^-1^). Plants were treated with T22 and/or inoculated with CMV, according to one of the following six conditions (25 plants for each condition): untreated and healthy control plants (PA); plants only treated with T22 (PB); plants only inoculated with CMV (PC); plants first treated with T22, and after 7 days inoculated with CMV (PD); plants simultaneously treated and inoculated with T22 and CMV (PE); and plants first inoculated with CMV, and after 7 days treated with T22 (PF). Fourteen days after CMV inoculation (i.e., when plants were 1-month-old) and until the plants were 3 months of age, tissues were collected and used for the following analyses.

### Evaluation of the Effect of T22 on the Severity of CMV Infection

When plants were 1- and 3-months-old, disease severity was measured using a 0–10 point rating scale, according to Murphy et al. (2003): 0 = no symptoms; 2 = mild mosaic symptoms on leaves; 4 = severe mosaic symptoms on leaves; 6 = mosaic and deformation of leaves; 8 = severe mosaic and deformation of leaves; 10 = severe mosaic and deformation of leaves with stunted growth. The disease severity values were expressed as the mean of 25 samples in each condition.

### CMV Detection by Enzyme-Linked Immunosorbent Assay (ELISA)

Samples of leaves, derived from four individual plants randomly chosen from the 25 plants in each condition, were collected when plants were 1- and/or 3-months-old. Double-antibody sandwich (DAS) Enzyme-Linked Immunosorbent Assay (ELISA) was conducted as follow: plates were coated with 200 μl per well of specific anti-CMV primary antibody (Bioreba, Reinach, Switzerland) diluted 1:1000 in a coating buffer (Bioreba), and then incubated for 2 h at 37°C. The plates were washed three times with PBS containing 0.05% Tween 20 (washing buffer). Crude plant extracts, in an extraction buffer (Bioreba) (1:10 w:v), were added to each well (200 μl) and kept at 4°C overnight. The plates were washed three times with a washing buffer (Bioreba). Anti-CMV immunoglobulin conjugated to alkaline phosphatase was diluted 1:1000 in a conjugate buffer (Bioreba) and added to the plates (200 μl per well). The plates were incubated for 2 h at 37°C, then washed three times with a washing buffer. P-nitrophenyl phosphate was dissolved in a substrate buffer (Bioreba) at 1 mg ml^-1^ and 200 μl were added to each well. The reaction was allowed to develop at room temperature in the dark. Absorbance at OD_405__nm_ was read by a microplate reader (Bio-Rad, model 550, Hercules, CA, USA). The mean absorbance value of four replicates for each sample was taken.

### Chlorophyll Content

At the end of the experimental trials, chlorophyll pigments were extracted in 10 ml of *N,N*-dimethylformamide from leaf disks (∅ 8 mm), taken from the central part of the leaves and derived from three individual plants randomly chosen from the 25 plants in each condition, and spectrophotometrically analyzed (model SP8001, Metertech, Taipei, Taiwan) at 647 and 664 nm. Chlorophyll *a* (Chl *a*) and chlorophyll *b* (Chl *b*) contents were calculated by the formulae reported by Moran (1982).

### Plant Growth

At the end of the experimental trial (3-month-old plants) plant growth parameters were considered. Plant height was monitored and dry matter (epigeous, DM) was obtained by drying the samples in a ventilated oven at 75°C until constant weight. In all cases, the value of three individual plants (randomly chosen from the 25 plants in each condition) was taken.

### Photosynthetic Gas Exchange

Measurements of photosynthetic activity (A), stomatal conductance to water vapor (*g*_s_) and intercellular CO_2_ concentration (*C*_i_) were carried out on 2- and 3-month-old plants in all six conditions, on apical mature and asymptomatic leaves belonging to three individual plants randomly chosen from the 25 plants in each condition. Before the analyses, selected plants were kept in a growth chamber at 28.4/18.1 ± 0.2°C (day/night) and photosynthetically active radiation (PAR) at plant height of 700–800 μmol photon m^-2^ s^-1^ provided by a mixture of high pressure sodium and metal halide lamps, during a 13 h photoperiod. Measurements were carried out using a LI-6400 portable photosynthesis system equipped with a 2 cm^2^ chamber and 6400–6440 LED light source, operating at a 380 ppm ambient CO_2_ concentration. The cuvette humidity and the temperature were kept constant during measurements in order to maintain constant air vapor pressure difference. Analyses were carried out between 12:00 and 14:00 h (solar time) under saturating light conditions (PAR about 1500 μmol photons m^-2^ s^-1^). Measurements were carried out maintaining the leaf temperature near to the air temperature in a temperature-controlled growth chamber (28–30°C).

### Antioxidant Enzyme Activities

Enzyme activities of superoxide dismutase (SOD, EC 1.15.1.1) and catalase (CAT, EC 1.11.1.6) were determined when plants were 1-month-old. Regarding SOD extraction, frozen leaves (1 g) belonging to three individual plants (randomly chosen from the 25 plants in each condition) were homogenized in 5 ml of cold 20 mM HEPES buffer, pH 7.2, containing 1 mM EGTA, 210 mM mannitol, and 70 mM sucrose, and then centrifuged at 1,500 × *g* for 5 min at 4°C. The pellet was discarded and the supernatant centrifuged again at 10,000 × *g* for 15 min at 4°C. The resulting supernatant contained cytosolic SOD (*cyt*-SOD) and the pellet mitochondrial SOD (*mit*-SOD). The mitochondrial pellet was homogenized in the cold buffer used previously. Both *cyt-*SOD and *mit*-SOD absorbance were monitored at 450 nm using a Bio-Rad model 550-microplate reader, according to the manufacturer’s instructions (Superoxide Dismutase Assay Kit, item No. 706002; Cayman Chemical, Ann Arbor, MI, USA). One unit of SOD was defined as the amount of enzyme needed to exhibit 50% dismutation of the superoxide radical.

Regarding CAT activity, frozen leaves (1 g) belonging to three individual plants (randomly chosen from the 25 plants in each condition) were homogenized in 5 ml of cold 50 mM potassium phosphate buffer, pH 7.0, containing 1 mM EDTA, and then centrifuged at 10,000 × *g* for 15 min at 4°C. The pellet was discarded and the resulting supernatant was used for the following steps. The absorbance of a product of the enzyme was read at 540 nm using a Bio-Rad model 550-microplate reader, according to the manufacturer’s instructions (Catalase Assay Kit, item No. 707002; Cayman Chemical). One unit of CAT was defined as the amount of enzyme needed for the formation of 1.0 nmol of formaldehyde per minute at 25°C.

### Phytohormone Bioassays

Ethylene, abscisic acid, salicylic acid, and jasmonic acid measurements were taken in leaves and roots belonging to three 1-month-old plants randomly chosen from the 25 plants in each condition. Regarding ET production, 15 min after excision [leaves were cut a few millimeters below the petiole, and 12–16 root tips were cut in small pieces (5 mm)], the material was enclosed in air-tight containers (250 ml). Gas samples (2 ml) were taken from the headspace of containers after 1 h incubation at 22°C. ET concentration was measured by a gas chromatograph (HP5890, Hewlett-Packard, Ramsey, MN, USA) using a flame ionization detector (FID), a stainless steel column (150 cm × 0.4 cm internal diameter packed with Hysep T) and detector temperatures of 70°C and 350°C, respectively, and a N_2_ carrier gas at a flow rate of 30 ml min^-1^ (Pellegrini et al., 2013). Quantification was performed against an external standard.

Abscisic acid was determined according to Perata et al. (1997). Frozen foliage and root samples (150 mg) were homogenized in 0.8 ml of 100% HPLC-grade water and incubated overnight at 4°C. After sonication and centrifugation (16,000× *g* for 10 min at 4°C), the supernatant was filtered through 0.2 μm Minisart SRT 15 filters and immediately analyzed. HPLC separation was performed at room temperature with a reverse-phase Dionex column (Acclaim 120, C18, 5 μm particle size, 4.6 mm internal diameter × 150 mm length). The compound was eluted using 70% solvent A (0.05 M acidified water) and 30% solvent B (methanol) for the first 6 min, followed by a 2 min linear gradient to 50% solvent B, 18 min with 50% solvent B, followed by 2 min linear gradient to 100% solvent B, 2 min with 100% solvent B followed by 2 min linear gradient to 70% solvent A. The flow-rate was 1 ml min^-1^. ABA was detected at the absorbance at 254 nm. To quantify the ABA content, known amounts of pure standard were injected into the HPLC system and an equation was formulated, correlating the peak area to ABA concentration.

Conjugated and free SAs were determined according to Pellegrini et al. (2013) with some modifications. Frozen foliage and root samples (150 mg) were added to 1 ml of 90% (v/v) methanol, vortexed and sonicated for 3 min. After centrifugation at 10,000 × *g* for 10 min at room temperature, the supernatant was transferred and the pellet was re-extracted in 0.5 ml of 100% (v/v) methanol followed by sonication and centrifugation, as described above. Supernatants from both extractions were combined and evaporated at 40°C under vacuum. The residue was resuspended in 0.25 ml of 5% (w/v) TCA and partitioned twice by using 0.8 ml of a 1:1 (v/v) mixture of ethyl acetate/cyclohexane. The upper phase containing free SA was concentrated at 40°C under vacuum and the lower aqueous phase (with conjugated SA) was hydrolyzed by adding 0.3 ml of 8 M HCl and incubating it for 60 min at 80°C. Both of the SAs collected from the upper phase and recovered from the lower phase were combined and dissolved in 600 μl of the mobile phase, containing 0.2 M sodium acetate buffer, pH 5.5 (90%) and methanol (10%). HPLC separation was performed at room temperature with the Dionex column described above. SA was quantified fluorimetrically (RF 2000 Fluorescence Detector, Dionex, Sunnyvale, CA, USA), with excitation at 305 nm and emission at 407 nm and was eluted using the mobile phase described above. The flow-rate was 0.8 ml min^-1^. To quantify the SA content, known amounts of pure standard were injected into the HPLC system and an equation was formulated, correlating the peak area to the SA concentration.

Jasmonic acid was determined according to Pellegrini et al. (2013). Frozen foliage and root samples (150 mg) were added to 1 ml of ethyl acetate and incubated overnight at 4°C. The extract was centrifuged at 10,000 × *g* for 10 min at 4°C. After adding a mixture of 0.2% (v/v) acidified water, the aqueous phase was filtered and immediately analyzed with HPLC using the Dionex column described above. JA was detected at the absorbance at 210 nm. The flow-rate was 1 ml min^-1^. To quantify the JA content, known amounts of pure standard were injected into the HPLC system and an equation was formulated, correlating peak area to JA concentration.

### Statistical Analysis

A minimum of three plants per ecophysiological/biochemical analysis were randomly chosen from the 25 plants in each condition of the two repeated experiments. Following the Shapiro–Wilk *W* test, data were analyzed using repeated measures (in the case of the measurements carried out for two time-points, i.e., ecophysiological parameters and disease severity) or one- and two-way analysis of variance (ANOVA). Comparison among means was determined by using Fisher’s least significant (LSD) test (*P* ≤ 0.05). All analyses were performed by NCSS 2000 Statistical Analysis System software (NCSS, Kaysville, UT, USA; see Hintze, 2001).

## Results

### CMV Infection Severity and Accumulation

**FIGURE 1:**
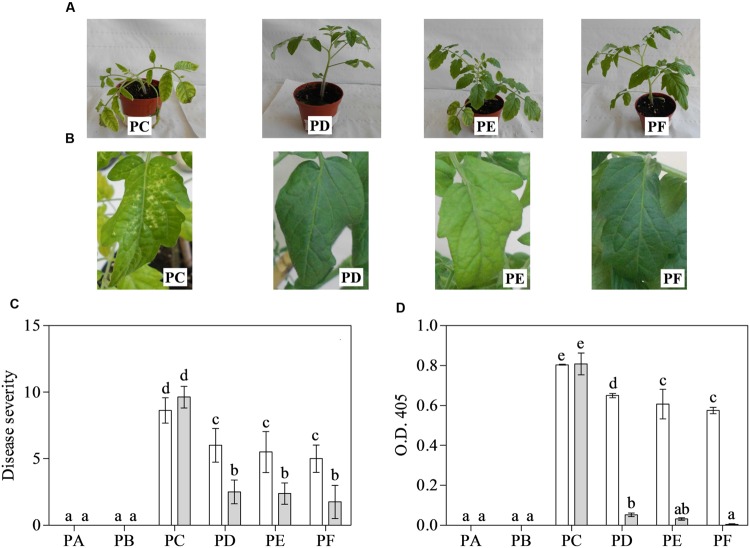
**Disease severity (0–10 scale) induced by *Cucumber mosaic virus* (CMV) and its accumulation in one- (white bars) and 3-month-old (grey bars) plants of *Solanum lycopersicum* var. *cerasiforme* treated, or not, with *Trichoderma harzianum* T-22. (A,B)** A representative entire plant or leaf of 1- or 3-month-old tomatoes, respectively. **(C)** Mean disease severity rating (*n* = 25) and **(D)** mean absorbance at OD_405__nm_ (*n* = 4) obtained by ELISA test. Bars represent standard errors of the means. Different letters indicate significant differences (*P* ≤ 0.05) among treatments and time evaluated by repeated measures **(C)** and two-ways **(D)** ANOVA. PA, healthy control; PB, plants treated with T22; PC, plants inoculated with CMV; PD, plants treated with T22 and, a week later, inoculated with CMV; PE, plants simultaneously treated and inoculated with T22 and CMV; PF, plants inoculated with CMV and, a week later, treated with T22.

Fourteen days after inoculation, PC always showed severe mosaic and deformation of leaves, and often stunting (**Figure 1A**). These symptoms were retained in the 3-month-old plants, where leaves also showed the typical necrosis induced by CMV-Fny (**Figure 1B**). The treatment with T22 always led to a significant modulation of symptoms, in all three conditions (PD, PE, and PF), above all when plants were 3-months-old. **Figure 1C** shows the disease severity. The interaction between treatment and time was significant, as well as the separate factors (according to the repeated measures ANOVA, *P* < 0.001). PC had an average significantly higher disease severity than plants also treated with T22 in the various combinations, especially in older plants (–74, –75 and –82%, in PD, PE, and PF, respectively), and also in the 1-month-old PF plants (about 2-fold lower than PC) (**Figure 1C**).

*Cucumber mosaic virus* accumulation in systemically infected leaves was measured by ELISA (**Figure 1D**). According to two-way ANOVA, the interaction between treatment and time was significant, as well as separate factors (*P* < 0.001). The younger plants, those inoculated with CMV and also treated with T22 showed significant differences in the mean absorbance value with respect to PC, above all in PE and PF (–24 and –29%, respectively). Furthermore, when plants were tested at 3 months of age, mean absorbance values for PD, PE, and PF were significantly much lower than those of PC (15-, 26-, and 170-fold, respectively). However, no significant difference was observed among 3-month-old PE and PF plants, nor between the PA and PB plants (**Figure 1D**). Healthy controls, as well as plants only exposed to T22, were symptomless and negative to CMV detection by ELISA, irrespective of leaf age.

### Chlorophyll Measurements

**FIGURE 2:**
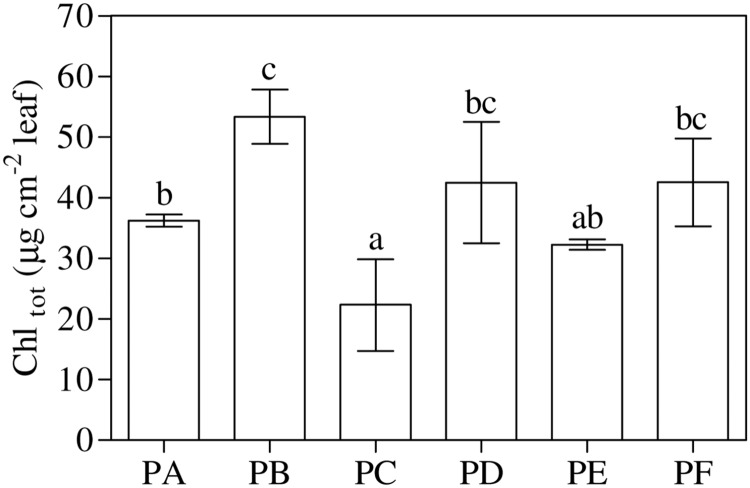
**Total chlorophyll (Chl_tot_) content in 3-month-old plants of *Solanum lycopersicum* var. *cerasiforme* infected, or not, by CMV and treated, or not, with *Trichoderma harzianum* T-22.** Bars represent standard errors of the mean values (*n* = 3). Different letters indicate significant differences (*P* ≤ 0.05) among treatments evaluated by one-way ANOVA. PA, healthy control; PB, plants treated with T22; PC, plants inoculated with CMV; PD, plants treated with T22 and, a week later, inoculated with CMV; PE, plants simultaneously treated and inoculated with T22 and CMV; PF, plants inoculated with CMV and, a week later, treated with T22.

As shown in **Figure 2** and Supplementary Table 1, total chlorophyll content, Chl *a* and Chl *b* showed the highest values with T22 alone and the lowest values with CMV alone. According to one-way ANOVA (*P* = 0.001), treatments with combined T22 and CMV (PD, PE, and PF) did not appeared to be statistically different from PA.

### Plant Growth

**FIGURE 3:**
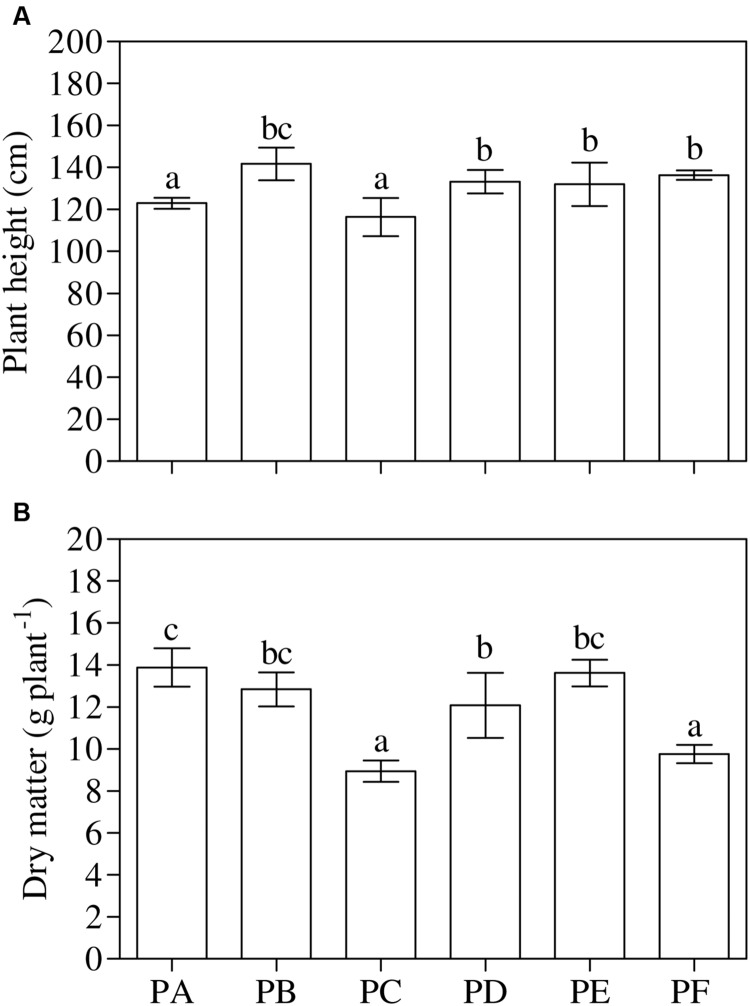
**Growth parameters measured in 3-month-old plants of *Solanum lycopersicum* var. *cerasiforme* infected, or not, by CMV, and treated, or not, with *Trichoderma harzianum* T-22. (A)** Plant height and **(B)** aboveground dry matter (*n* = 3). Different letters indicate significant differences (*P* ≤ 0.05) among treatments evaluated by one-way ANOVA. Bars represent standard errors of the means. PA, healthy control; PB, plants treated with T22; PC, plants inoculated with CMV; PD, plants treated with T22 and, a week later, inoculated with CMV; PE, plants simultaneously treated and inoculated with T22 and CMV; PF, plants inoculated with CMV and, a week later, treated with T22.

One-way ANOVA (*P* < 0.001) highlighted that the treatment with T22 always led to a significant increase in height with respect to PA and PC, in all the three conditions - PD, PE, and PF - (about 1.2-fold higher than PA), and above all when they were first inoculated with CMV and then treated with T22 (PF, +11%), as shown in **Figure 3A**. On the other hand, epigeous DM was not influenced by the T22 treatment (**Figure 3B**). One-way ANOVA (*P* < 0.001) showed that in plants treated with T22 alone and subsequently, or simultaneously, inoculated with CMV, DM was not statistically different from PB plants. Plants inoculated with CMV, alone or before T22 treatment, showed significantly lower DM values than those of PD (PC vs. PD, –35%, and PF vs. PD, –24%) and PE treatments (PC vs. PE, –52%, and PF vs. PE, –39%). In PC and PF, a mean of 33% in the reduction of epigeous DM accumulation was observed in comparison with PA.

### Photosynthetic Gas Exchange

Concerning A values, the interaction between treatment and time was significant (*P* = 0.043), as well as separate factors (*P* < 0.001, treatment, and *P* = 0.002, time) (according to the repeated measures ANOVA, **Table 1**). In 2-month-old plants, the most significant increase in A was observed in PE compared with PA (+17%). Lowest A values were detected in plants infected with CMV alone, regardless of plant age. After 3 months, a general reduction in A values was observed in all the treatments, due to the beginning of leaf senescence, except for PC and PD. For these older plants, a higher A was measured in PB, PD, and PE, while PF showed the same behavior as PA. The average value of A in PD, PE, and PF 3-month-old plants was significantly higher than that measured in PC (+59, +60, and +33%, respectively). Concerning *g*_s_ values, the interaction between treatment and time was significant (*P* = 0.039), as well as separate factors (*P* = 0.001, treatment, and *P* = 0.041, time). In the 2-month-old PC, *g*_s_ was very low (about –40% in comparison with PA and PB).

**Table 1:** Leaf gas exchange parameters measured in 2- and 3-month-old plants of *Solanum lycopersicum* var. *cerasiforme* infected or not by *Cucumber mosaic virus* (CMV), and treated or not with *Trichoderma harzianum* T-22.

	A (μmol CO_2_ m^-2^ s^-1^)		*g*_s_ (mol H_2_O m^-2^ s^-1^)		*C*_i_ (ppm)	
Age (months)	2	3	2	3	2	3
PA	20.0 ± 0.55 def	14.4 ± 0.65 b	0.27 ± 0.025 b	0.39 ± 0.019 e	254 ± 7.5 bc	300 ± 6.0 cd
PB	21.8 ± 1.45 fg	18.5 ± 0.60 de	0.26 ± 0.001 b	0.34 ± 0.016 d	236 ± 9.0 b	227 ± 41.5 b
PC	12.5 ± 1.20 ab	11.2 ± 0.25 a	0.15 ± 0.018 a	0.34 ± 0.083 de	156 ± 16.5 a	320 ± 12.0 d
PD	17.6 ± 1.70 cd	17.8 ± 0.85 d	0.31 ± 0.022 c	0.34 ± 0.081 de	267 ± 8.0 bcd	279 ± 21.2 cd
PE	23.4 ± 0.65 g	17.9 ± 0.45 d	0.28 ± 0.007 bc	0.30 ± 0.058 bcd	245 ± 11.3 b	249 ± 14.7 b
PF	20.4 ± 2.00 ef	14.9 ± 0.10 bc	0.32 ± 0.040 d	0.29 ± 0.056 bc	241 ± 16.8 b	289 ± 19.1 cd

*For each parameter (within the pairs of columns), different letters indicate significant differences (*P* ≤ 0.05) among the treatments and the time evaluated by repeated measures ANOVA. Data are shown as mean ± standard error (*n* = 3). PA, healthy control; PB, plants treated with T22; PC, plants inoculated with CMV; PD, plants treated with T22 and, a week later, inoculated with CMV; PE, plants simultaneously treated and inoculated with T22 and CMV; PF, plants inoculated with CMV and, a week later, treated with T22. A, photosynthetic activity; *g*_*s*_, stomatal conductance to water vapor; *C*_*i*_, intercellular CO_2_ concentration.*

After 3 months, a general increase in *g*_s_ was observed in all the treatments, with the exception of PF. Concerning *C*_i_ values, the interaction between treatment and time was significant (*P* = 0.022), as well as separate factors (*P* = 0.005, treatment, and *P* = 0.031, time). The lowest value of *C*_i_ was found in 2-month-old PC plants. All the other treatments were significantly higher and there were no differences between them. Conversely, in the 3-month-old plants, the *C*_i_ values were not statistically different to those of healthy control plants, except for PB and PE, which showed lower levels than PA (–24 and 17%, respectively) and PC (–29 and –22%).

### Enzyme Activities

**FIGURE 4:**
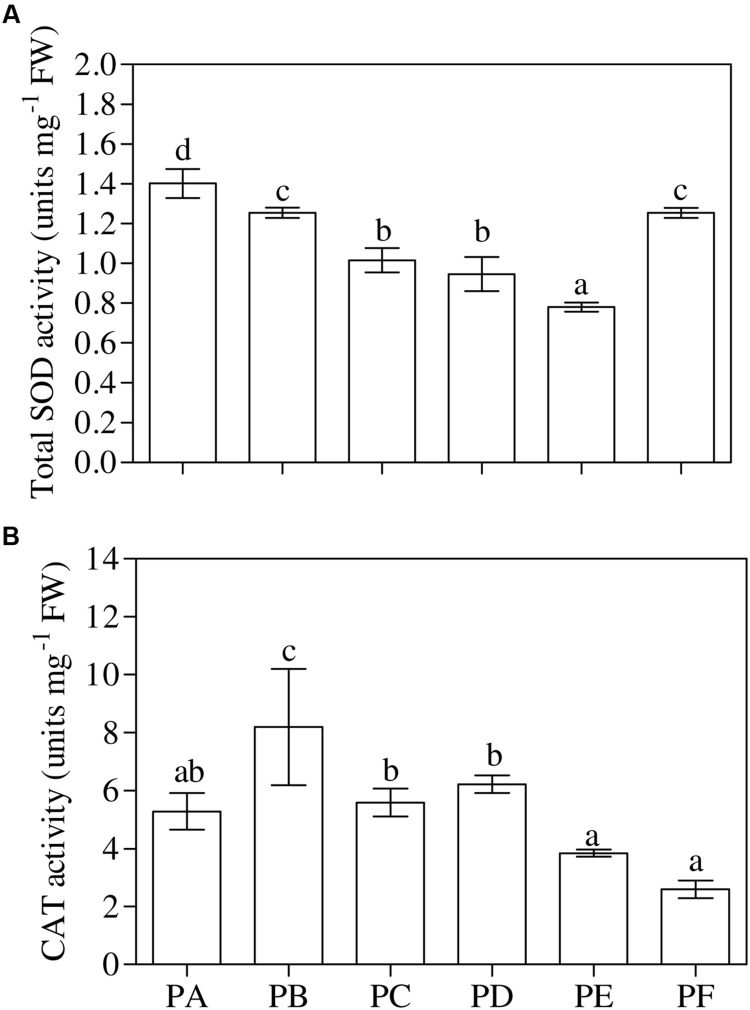
**(A)** Total superoxide dismutase (SOD) and **(B)** catalase (CAT) activity measured in 1-month-old *Solanum lycopersicum* var. *cerasiforme* infected, or not, by CMV, and treated, or not, with *Trichoderma harzianum* T-22. Bars represent standard errors of the mean values (*n* = 3). Different letters indicate significant differences (*P* ≤ 0.05) among treatments evaluated by one-way ANOVA. PA, healthy control; PB, plants treated with T22; PC, plants inoculated with CMV; PD, plants treated with T22 and, a week later, inoculated with CMV; PE, plants simultaneously treated and inoculated with T22 and CMV; PF, plants inoculated with CMV and, a week later, treated with T22.

One-way ANOVA (*P* < 0.001) revealed that the lowest values of total SOD activity were in PE and the highest in PA (**Figure 4A**). The values of total SOD activity in PF remained significantly lower, when compared to PA (–11%), but not significantly different from plants treated with T22 alone. The trend of *cyt*-SOD activity paralleled that of total SOD activity, except PB. On the other hand, *mit*-SOD activity was the lowest in the plants inoculated with CMV alone, the highest in plants treated only with T22, and intermediate in PD, PE, and PF, with values significantly lower than PA (–22, –28 and –11%, respectively) (Supplementary Table 2). One-way ANOVA (*P* < 0.001) highlighted that CAT activity was induced by the presence of T22 alone (+55% compared to PA), with clear and significant depressions in PF (–51%). PC, PD, and PE were not significantly different from the control significantly different from the control (**Figure 4B**).

### Phytohormone Content

**FIGURE 5:**
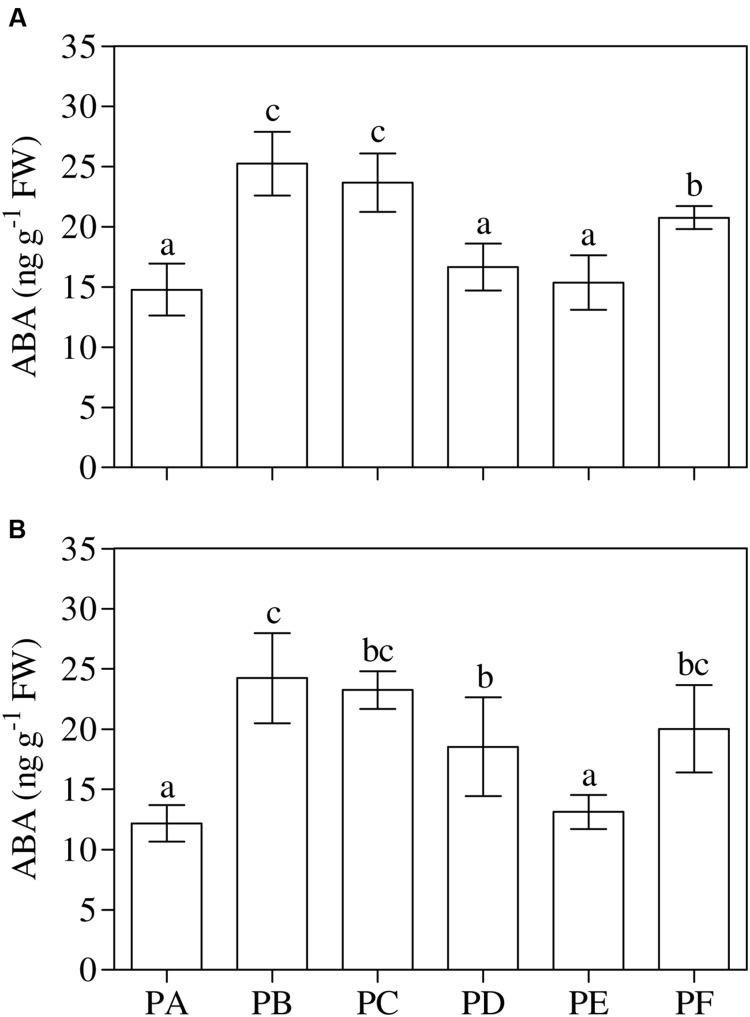
**(A,B)** Abscisic acid (ABA) content in leaves **(A)** and roots **(B)** of 3-month-old plants of *Solanum lycopersicum* var. *cerasiforme* infected, or not, by CMV, and treated, or not, with *Trichoderma harzianum* T-22. Bars represent standard errors of the mean values (*n* = 3). Different letters indicate significant differences (*P* ≤ 0.05) among treatments evaluated by one-way ANOVA. PA, healthy control; PB, plants treated with T22; PC, plants inoculated with CMV; PD, plants treated with T22 and, a week later, inoculated with CMV; PE, plants simultaneously treated and inoculated with T22 and CMV; PF, plants inoculated with CMV and, a week later, treated with T22.

**FIGURE 6:**
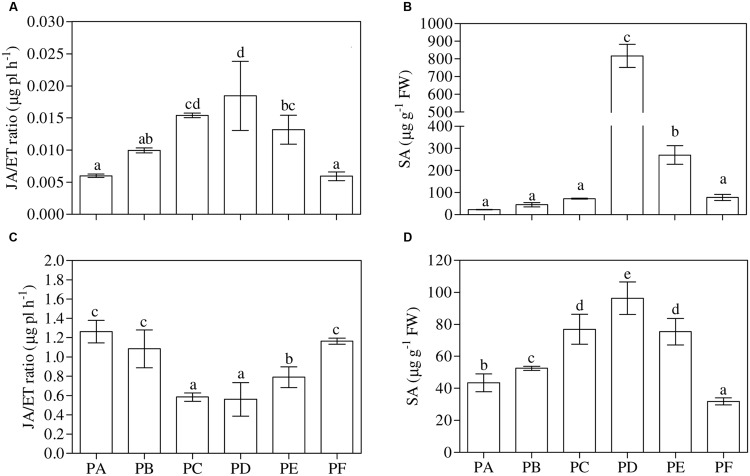
**(A,B)** Jasmonic acid/ethylene ratio (JA/ET) and total salicylic acid concentrations (SA) in leaves and **(C,D)** in roots of 3-month-old plants of *Solanum lycopersicum* var. *cerasiforme* infected, or not, by CMV, and treated, or not, with *Trichoderma harzianum* T-22. Bars represent standard errors of the mean values (*n* = 3). Different letters indicate significant differences (*P* ≤ 0.05) among treatments evaluated by one-way ANOVA. PA, healthy control; PB, plants treated with T22; PC, plants inoculated with CMV; PD, plants treated with T22 and, a week later, inoculated with CMV; PE, plants simultaneously treated and inoculated with T22 and CMV; PF, plants inoculated with CMV and, a week later, treated with T22.

One-way ANOVA (*P* < 0.001) showed that the treatment with T22 (PB) or CMV (PC) led to a significant increase in the leaf ABA content when applied alone (+71 and +60% compared to PA, respectively), and when the plants were first inoculated with CMV (PF, +40%) (**Figure 5A**). The same behavior of ABA content was found in the roots, except for PD (+52%) (one-way ANOVA, *P* = 0.001) (**Figure 5B**). One-way ANOVA (*P* < 0.001) revealed that the foliar JA/ET ratio significantly increased with CMV alone (2.5-fold higher than PA) (**Figure 6A**). Regarding the combined treatments, JA/ET was significantly higher in PD and PE (3- and 2-fold higher, respectively). One-way ANOVA (*P* < 0.001) demonstrated that the lowest values of total leaf SA content were in PA, PB, PC, and PF. T22 led to a significant increase when the plants were treated before or simultaneously with CMV inoculation (about 36- and 12-fold higher than PA) (**Figure 6B**). The trends of conjugated and free SA content paralleled that of total SA (Supplementary Table 3). One-way ANOVA (*P* < 0.001) showed that the root JA/ET ratio (**Figure 6C**) significantly decreased with CMV alone (about 2-fold lower than PA). Regarding the combined treatments, JA/ET decreased more in PD and PE than PA (–56 and –37%, respectively). One-way ANOVA (*P* < 0.001) highlighted that the lowest values of total root SA content were found in PF (–27% compared to PA) and the highest in PD (+122%) (**Figure 6D**). This concentration also increased in PB, PC, and PE (+21, +77, and +73%, respectively). Generally, the trends of the free root SA content paralleled that of total SA content. The root conjugated SA concentration was lowest in the plants treated with T22 after the CMV inoculation (PF) and not significantly different from PA and PC. T22 caused a significant increase when the plants were treated before or simultaneously with the CMV inoculation (PD and PE, about 2-fold higher than PA) (Supplementary Table 3).

## Discussion

The *Trichoderma* species are considered to be one of the most versatile BCAs (Mukherjee et al., 2013), and have long been used for managing plant infections sustained by pathogenic fungi (Weindling, 1934; Wells, 1988; Vinale et al., 2009) and bacteria (Segarra et al., 2009). Recently, the use of the T22 strain of *T. harzianum* was also investigated as a new approach for controlling viruses, considering that chemical treatments have no effect on these pathogens (Vitti et al., 2015). Some of our previous studies were based on the use of T22 for stimulating the induction of tomato defense responses against CMV. It was demonstrated that the action of T22 involves the modulation of viral symptoms, with the inhibition of the RNA-dependent RNA polymerase gene, and the involvement of ROS as secondary messengers of the defense response against the virus (Vitti et al., 2015). To the best of the authors’ knowledge, the present study provides new information regarding the changes occurring in the three-cross-talk tomato-CMV-T22 at the photosynthetic, antioxidant and phytohormone systems levels.

The present results confirm that T22 is able to protect tomato plants against CMV not only in terms of a modulation of symptoms, and thus reducing the disease severity, but also in terms of a reduction in foliar viral titer. The observation of symptoms, as well as the disease severity, demonstrated that treatment with T22 reduced CMV in all plants until its suppression, with a more marked effect in 3-month-old plants.

The ELISA data highlight that the effect of T22 was particularly clear when it was applied after CMV inoculation (PF). Counteracting the spread of symptoms, T22 prevents a reduction in green photosynthetic tissues and the degradation of pigments, which has been observed in plants infected by CMV (PC) and in other pathosystems (Silveira et al., 2015).

One of the effects of T22 application is an increase in plant growth, also in plants under stress conditions (Harman, 2000; Shoresh et al., 2010), indirectly due to its strong anti-pathogenic activity, and directly through the (i) induction of the biosynthesis of phytohormones, (ii) improved solubilization and uptake of soil nutrients, (iii) root hardening and development, (iv) enhancement in the rate of carbohydrate metabolism, and (v) photosynthesis increase (Sofo et al., 2012; Stewart and Hill, 2014).

The plant growth-promoting effect of T22 was described for the first time by Windham et al. (1986) for the case of tomato and tobacco. The increase in plant growth also depends on the species and genotype involved and, in some cases, the inconsistency in boost growth may be related to differences in growth conditions (Tucci et al., 2011; Stewart and Hill, 2014). In our case, the T22-induced growth seemed to lead to some improvements in CMV-infected plants, since plant height was significantly enhanced by T22 application. The epigeous DM accumulated in plants treated with T22 alone (PB) did not differ significantly between PA, PD, and PE. However, there was significant reduction in the plants inoculated only with CMV (PC) or when the virus infected the plants before treatment with T22 (PF). Whereas the effect of T22 in this combination was not significant on plant height (in comparison to PB), it could (i) form nutrient interaction, (ii) affect the availability of nutrients which are essential for growth (Benítez et al., 2004) and consequently, (iii) act in the reallocation of various physiological activities from vegetative growth toward defense activation (Gruner et al., 2013). Our data highlighted that T22 improved photosynthesis: the values of A and the total Chl content were the same or higher in all plants treated with T22 and inoculated with CMV (PD, PE, and PF) than PA, regardless of plant age. This finding is in agreement with much evidence that *Trichoderma* strains are able to increase photosynthetic rates and efficiency in plants (Vargas et al., 2009; Mastouri et al., 2010; Shoresh et al., 2010), mainly through improving the redox status of the plant (Harman, 2011). During the symbiotic interaction, the enzymatic activity in the fungal cells affect the sink activity of roots, thereby moving carbon partitioning toward roots and increasing the rate of leaf photosynthesis (Vargas et al., 2009).

In order to explain the improving effect of fungal symbiosis on plant assimilation, it has been hypothesized that symbioses could stimulate the rates of photosynthesis (Kaschuk et al., 2009). In our case, CMV infection (PC) significantly reduced photosynthesis, in accordance with other authors (van Kooten et al., 1990; Rahoutei et al., 2000), by a decreased stomatal conductance (as observed in 2-month-old plants) which can regulate plant defenses against invaders especially at the early stages of infection, as reported by Alazem and Lin (2015). Thus, the depressed photosynthetic activity was mainly caused by stomatal limitation, but also by non-stomatal inhibition in 3-month-old PC (as confirmed by unchanged *C*_i_ values), as already reported in other pathosystems (e.g., Lorenzini et al., 1997; Zhou et al., 2004; Bermúdez-Cardona et al., 2015).

As reported by Vitti et al. (2015), CMV infection leads to the generation of ROS - especially H_2_O_2_ - which can react with proteins, lipids and deoxyribonucleic acid causing oxidative damage and impairing the normal functions of plant cells. In order to overcome these effects, plants develop antioxidant defense systems comprising both enzymatic and non-enzymatic components which (i) prevent ROS accumulation, and (ii) alleviate oxidative damage occurring during the infection (Lehmann et al., 2015).

Superoxide dismutase is the most important enzyme in the defense mechanism, which catalyzes the dismutation of superoxide into O_2_ and H_2_O_2_ (Gill and Tuteja, 2010). In our experiments, CMV, alone or in combination with T22 in PD and PE, caused a significant depression of total SOD activity, which may lead to the induction of defense responses according to several studies (Alscher et al., 2002; Battistoni, 2003; Broxton and Culotta, 2016). By contrast, the highest *mit*-SOD activity was in plants treated with T22 alone, suggesting that this enzyme plays a pivotal role in ROS detoxification preventing oxidative damage and protecting the photosynthetic apparatus. CAT scavenges the toxic and unstable ROS and converts them into less toxic and more stable components, such as O_2_ and water (Gill and Tuteja, 2010). CAT accumulation was reported in plants treated with T22 alone which suggests that this enzyme can increase cell wall resistance and act as a signal for the induction of defensive genes. By contrast, the absence of any enhancement (as observed in PC, PD, and PE) of CAT activity suggests that this enzyme could interact with the virus (especially some virus elements, such as CMV 2b protein, Inaba et al., 2011; Mathioudakis et al., 2013), thus facilitating the persistence of H_2_O_2_ in the cell which, in turn, can induce SA accumulation (León et al., 1995), as confirmed by the significant increase of free and conjugated SA levels in PD and PE, respectively. We can speculate that 2b protein of CMV (known to be a viral RNA silencing suppressors and also involved in viral movement and symptom induction, Goto et al., 2007) did not sequester CAT in cells in favor of viral infection, but it can bind to catalase genes (for example catalase3, Masuta et al., 2012) inducing a specific defense mechanism. SA, JA, and ET play a crucial role in plant disease and pest resistance. Induced resistance responses in plants are subdivided to two major categories: systemic acquired resistance (SAR) and induced systemic resistance (ISR). SAR is associated with both local and systemic increases in SA levels, and the expression of pathogenesis-related genes is mediated via the SA pathway. ISR does not depend on the accumulation of SA or pathogenesis-related proteins, but on pathways regulated by JA and ET (Elsharkawy et al., 2013). Evidence indicating the partial involvement of the SA-dependent signaling pathway in ISR has been reported (Elsharkawy et al., 2012). In our experiments, when T22 was applied before or simultaneously with CMV (PD and PE), it triggered concomitant marked increases in the foliar JA/ET ratio and SA levels, compared to healthy PA controls, suggesting that ISR and SAR have occurred. The concomitant mild increase in H_2_O_2_ content, as revealed by Vitti et al. (2015), confirms that these compounds constitute a self-amplifying system, where JA and SA enhance H_2_O_2_ levels which could interact with CAT activity (as confirmed by unchanged CAT activity levels), in accordance with Chen et al. (1993).

T22 treatment subsequent to CMV infection (PF) did not induce SAR and ISR, but seemed to stimulate an ABA-related resistance, as confirmed by the absence of any alteration in the JA/ET ratio and SA levels and by the significant increase in ABA levels in leaves and in roots, with respect to PA plants. The JA/ET ratio and SA levels in roots suggest that the T22 treatment induced SAR in PD and PE combinations. This is not surprising, since an extensive survey comparing shoot and root chemical defenses corroborated the fact that shoots and roots have a distinct defense mechanism (Balmer and Mauch-Mani, 2013). Although most of the compounds in roots are also found in shoots, the constitutive levels could be highly divergent, as in our case.

Therefore, regarding the role of plant hormones in the regulation of the tomato-CMV-T22 complex, it is possible to conclude that in all plants treated with T22 and inoculated with CMV (i) the responsiveness to ABA, JA, ET, and SA was required in order to develop lower levels of disease, and (ii) a cross-talk between different hormonal-related signaling pathways had occurred. The significant increase in the foliar JA/ET ratio, as well as the SA levels observed in both leaves and roots of PD and PE, suggest that T22 was able to induce systemic resistance against CMV, requiring not only the JA/ET, but also the SA signaling pathway, when T22 was used before or simultaneously to CMV. In agreement with Martínez-Medina et al. (2010), these findings indicate that the protective effect against CMV conferred on tomato by T22 was not due to direct antagonism, but was a plant-mediated phenomenon. In fact, the absence of any enhancement (observed in PD and PE in comparison with PC) of JA/ET ratio suggests that the CMV infection is self-limiting due to self-activation of ISR.

## Conclusion

Data described in this paper demonstrate that early treatment with *T. harzianum* T-22 is able to induce systemic defense responses against CMV-Fny in *S. lycopersicum* var. *cerasiforme* by interacting with plant hormones. Furthermore, T22 is able to improve the photosynthetic performance and promote growth. In conclusion, our data indicate that the T22-based strategy is a largely practicable way to pursue the goal of an effective measure against CMV.

## Author Contributions

The work presented here was carried out in collaboration among all authors. SL, AdS, AnS, and MN defined the research theme and obtained funding. AV, EP, SL, AdS, MV, and MN designed methods, carried out laboratory experiments, and analyzed the data. AV, EP, CN, SL, AdS, and MN co-designed experiments, discussed analyses, interpreted the results and wrote the paper. All authors have contributed to discuss the results and implications of the work and to comment on the manuscript at all stages before approvation.

## Conflict of Interest Statement

The authors declare that the research was conducted in the absence of any commercial or financial relationships that could be construed as a potential conflict of interest.
