# Supplementary material for: Trichoderma harzianum T-22 Induces Systemic Resistance in Tomato Infected by Cucumber mosaic virus
Source: Front Plant Sci. 2016 Oct 10;7:1520. doi: 10.3389/fpls.2016.01520 (PMC5056173; doi:10.3389/fpls.2016.01520)
Supplement: Supplementary file 1 [file Table_1.docx]

**Supplemental Material**

Table 1. Chlorophyll *a* (Chl *a*) and chlorophyll *b* (Chl *b*) content measured in three-month-old plants of *Solanum lycopersicum* var. *cerasiforme* infected, or not, by *Cucumber mosaic virus*, and treated, or not, with *Trichoderma harzianum* T-22. Mean values (*n* = 3) ± SE with different letters are significantly different (*P* ≤ 0.05). PA, healthy control; PB, plants treated with T22; PC, plants inoculated with CMV; PD, plants treated with T22 and, a week later, inoculated with CMV; PE, plants simultaneously treated and inoculated with T22 and CMV; PF, plants inoculated with CMV and, a week later, treated with T22.

|  | Chl *a* | Chl *b* |
| --- | --- | --- |
|  | (µg cm^-2^ leaf) | |
| PA | 27.5±0.94 b | 8.7±0.67 b |
| PB | 39.4±3.30 c | 14.0±1.21 d |
| PC | 16.3±5.79 a | 6.0±1.79 a |
| PD | 30.4±9.14 bc | 12.0±1.10 cd |
| PE | 23.4±0.54 ab | 8.8±0.30 b |
| PF | 31.3±5.64 bc | 11.2±1.63 c |
