# Supplementary material for: Trichoderma harzianum T-22 Induces Systemic Resistance in Tomato Infected by Cucumber mosaic virus
Source: Front Plant Sci. 2016 Oct 10;7:1520. doi: 10.3389/fpls.2016.01520 (PMC5056173; doi:10.3389/fpls.2016.01520)
Supplement: Supplementary file 2 [file Table_2.docx]

**Supplemental Material**

Table 2. Cytosolic and mitochondrial superoxide dismutase (SOD) activity measured in one-month-old plants of *Solanum lycopersicum* var. *cerasiforme* infected, or not, by *Cucumber mosaic virus*, and treated, or not, with *Trichoderma harzianum* T-22. Mean values (*n* = 3) ± SE with different letters are significantly different (*P* ≤ 0.05). PA, healthy control; PB, plants treated with T22; PC, plants inoculated with CMV; PD, plants treated with T22 and, a week later, inoculated with CMV; PE, plants simultaneously treated and inoculated with T22 and CMV; PF, plants inoculated with CMV and, a week later, treated with T22.

|  | Cytosolic SOD | Mitochondrial SOD |  |
| --- | --- | --- | --- |
|  | (units mg^-1^ FW) | | |
| PA | 0.62±0.051 d | 0.78±0.022 d |  |
| PB | 0.36±0.047 b | 0.90±0.021 e |  |
| PC | 0.54±0.023 c | 0.48±0.038 a |  |
| PD | 0.34±0.023 b | 0.61±0.062 b |  |
| PE | 0.22±0.018 a | 0.56±0.006 b |  |
| PF | 0.56±0.048 cd | 0.69±0.035 c |  |
