# Supplementary material for: Trichoderma harzianum T-22 Induces Systemic Resistance in Tomato Infected by Cucumber mosaic virus
Source: Front Plant Sci. 2016 Oct 10;7:1520. doi: 10.3389/fpls.2016.01520 (PMC5056173; doi:10.3389/fpls.2016.01520)
Supplement: Supplementary file 3 [file Table_3.docx]

**Supplemental Material**

Table 3. Conjugated and free salicylic acid concentrations in leaves and roots of one-month-old plants of *Solanum lycopersicum* var. *cerasiforme* infected, or not, by *Cucumber mosaic virus*, and treated, or not, with *Trichoderma harzianum* T-22. Mean values (*n* = 3) ± SE with different letters are significantly different (*P* ≤ 0.05). PA, healthy control; PB, plants treated with T22; PC, plants inoculated with CMV; PD, plants treated with T22 and, a week later, inoculated with CMV; PE, plants simultaneously treated and inoculated with T22 and CMV; PF, plants inoculated with CMV and, a week later, treated with T22.

|  | Leaves | | Roots | |
| --- | --- | --- | --- | --- |
|  | Conjugated form | Free form | Conjugated form | Free form |
|  | (µg g^-2^ FW) | | | |
| PA | 11.5±1.43 a | 11.3±1.49 a | 15.6±0.93 ab | 27.9±4.63 b |
| PB | 9.1±0.76 a | 35.8±9.82 a | 16.3±1.30 b | 36.2±2.46 c |
| PC | 9.6±1.78 a | 61.8±1.87 a | 15.5±0.57 ab | 61.4±8.82 d |
| PD | 44.6±6.69 c | 772.6±71.7 c | 26.7±1.89 c | 69.7±8.77 d |
| PE | 30.9±8.67 b | 239.0±35.1 b | 32.4±1.01 d | 43.0±8.21 c |
| PF | 10.9±1.08 a | 67.0±13.6 a | 14.3±0.51 a | 17.6±2.01 a |
